# Efficacy and Safety of Combination Androgen‐Receptor Signaling Inhibitors, Denosumab, and Local Radiotherapy for Poly‐Metastatic Prostate Cancer

**DOI:** 10.1002/cnr2.70355

**Published:** 2025-10-01

**Authors:** Makoto Kawase, Kota Kawase, Yuki Tobisawa, Koji Iinuma, Keita Nakane, Takuya Koie

**Affiliations:** ^1^ Department of Urology Gifu University Graduate School of Medicine Gifu Japan

**Keywords:** androgen receptor signaling inhibitor, denosumab, local radiation therapy, metastasis‐directed therapy, metastatic castration‐sensitive prostate cancer

## Abstract

**Background:**

The Treatment of Metastatic Castration‐Sensitive Prostate Cancer (mCSPC) has dramatically changed over the past decade. To improve oncological outcomes, pharmacological treatments have evolved to include two‐or three‐drug combinations, and the efficacy of local radiation therapy (LRT) at the prostate combined with denosumab chemotherapy has been discussed.

**Aims:**

This study aimed to provide an interim evaluation of the combination of androgen receptor signaling inhibitors denosumab, LRT, and metastasis‐directed therapy (MDT) for prostate cancer with multiple bone metastases (poly‐PCa).

**Methods:**

We are currently conducting a single‐arm prospective study to evaluate the combination of enzalutamide, LRT, denosumab, and MDT in terms of improving oncological outcomes in patients with poly‐PCa. The primary endpoints were prostate‐specific antigen (PSA)‐based progression‐free survival (PFS) and radiographic PFS (rPFS).

**Results:**

Twenty patients have been enrolled in the study to date. The median follow‐up period thus far is 18.0 months (interquartile range, 14.0–25.8 months). The 1‐year PFS rate is 84.4% at present, and the rPFS rate is 100%. Seventeen patients (85.0%) achieved a PSA reduction of ≥ 90% versus their baseline values at enrollment, and 10 (50.0%) maintained PSA levels of < 0.2 ng/mL.

**Conclusion:**

This treatment strategy is expected to improve the oncological outcomes of patients with poly‐PCa. A more comprehensive report of this study with more patients and a longer observation period is forthcoming.

## 1 Introduction

Although no substantial advances have been made in the treatment of metastatic castration‐sensitive prostate cancer (mCSPC), significant progress has been achieved over the past decade. Patient overall survival (OS) and quality of life (QOL) indicators have improved significantly with the availability of doublet combination therapies consisting of androgen receptor signaling inhibitors (ARSIs) and androgen deprivation therapy (ADT) [1]. The 3‐year OS rate for this malignancy has been reported to be relatively favorable at 80%. However, its 3‐year biochemical recurrence‐free survival (PFS) and radiographic PFS (rPFS) rates are both 67%, indicating that doublet therapy alone may have limited long‐term efficacy [1]. Triplet therapy with docetaxel, darolutamide, and ADT has shown survival benefits in prostate cancers (PCas) with large tumor volumes and high numbers of metastatic sites [2]. The STAMPEDE trial suggested that local radiation therapy (LRT) delivered to the prostate may improve OS in patients with PCa and newly identified oligometastases [3]. Conversely, the HORRAD trial revealed no efficacy for LRT in patients with PCa who had bone metastases, including 67% of those with bone metastases in ≥ 5 sites [4]. A Japanese cohort study suggested that LRT combined with standard pharmacological therapy for *de novo* mCSPC may extend survival and reduce symptomatic local events in patients with PCa, even those with a high tumor burden [5]. The PEACE‐1 study showed that a combination of ADT, ARSI, abiraterone, and RT was effective for treating patients with mCSPC [6]. The benefits of LRT include the prevention of serious genitourinary complications associated with PCa progression, such as urinary retention, pain, and hemorrhage [6]. Indeed, combination therapy including both ARSI and LRT has been reported to reduce the frequency of PCa‐related complications [6]. However, the efficacy of LRT for improving oncological outcomes remains unclear [6].

The efficacy of metastasis‐directed therapy (MDT) for PCa with multiple bone metastases (poly‐PCa) has not yet been clarified [3]. Although the STOMP trial reported the efficacy of MDT in metachronous oligometastatic PCa [7], no studies published in the literature thus far (to our knowledge) have demonstrated the efficacy of MDT in patients with poly‐PCa. As a treatment for patients with castration‐resistant PCa (CRPC) and bone metastases, denosumab has been shown to significantly delay skeletal‐related events compared with zoledronic acid, as well as have a favorable effect on pain and health‐related QOL in this patient group [8]. Even in patients with nonmetastatic CRPC, denosumab has been reported to significantly prolong the interval until bone metastases develop [8]. In this study, we aimed to provide an interim report on the efficacy of an ARSI, denosumab, LRT, and MDT combination treatment for treating poly‐PCa.

## 2 Methods

We are currently conducting a single‐arm prospective interventional study on the combination of enzalutamide, denosumab, LRT, and MDT to improve the oncological outcomes in patients with poly‐PCa. The study was approved by the Institutional Review Board of Gifu University (approval number 2021‐B041) and is registered with UMIN at the following link: https://center6.umin.ac.jp/cgi‐open‐bin/ctr/ctr_view.cgi?recptno=R000062255 (please note that the current number of registered cases is incorrect; this information will be corrected at a later date). Personal data is listed at https://center6.umin.ac.jp/ice/62255. However, access to this system is strictly regulated by personal identification and a password, and it is not accessible to the general public. For this study, we defined poly‐PCa as the presence of ≥ 4 bone metastases. The target number of patients registered was set at 38. The target sample size for this study was set at 38 patients based on a comparison with the 2‐year PFS rate of 77% reported in the ARCHES trial. In virtual simulations based on this sample size, the 95% confidence interval for 2‐year PFS was determined to be (63.2%, 89.5%). The research period was designated as December 2021 to December 2027. The patients who were enrolled in this trial met the following selection criteria: they were aged between 20 and 90 years old, had ≥ 4 bone metastases, were eligible for enzalutamide and denosumab, had an expected survival of ≥ 6 months, had Eastern Cooperative Oncology Group‐Performance Status ≤ 1, and had provided informed consent to participate in this trial. The exclusion criteria included patients with visceral metastases, excluding the lungs, those who refused LRT, those with osteonecrosis of the jaw or severe periodontal disease, and those with a history of chemotherapy. Patients with poly‐PCa, regardless of the presence or absence of lymph node metastasis, were treated with 160 mg of enzalutamide once daily, in addition to a luteinizing hormone‐releasing hormone antagonist and denosumab administered once every 4 weeks. Enzalutamide was selected as the ARSI for this trial because it binds directly to the ligand‐binding domain of the androgen receptor to inhibit androgen receptor rearrangement, deoxyribonucleic acid binding, and androgen receptor‐mediated transcription. It also rapidly decreases prostate‐specific antigen (PSA) levels [1]. A high incidence of rash has also been observed among Japanese patients with PCa who received apalutamide, and abiraterone has been reported to cause adverse events when used in conjunction with steroids [9, 10]. During the administration of these agents, PSA levels were measured at least every 3 months; and computed tomography, magnetic resonance imaging, and bone scintigraphy were performed at least every 6 months to assess the progression of regional or metastatic lesions. If the disease remained stable without progression for > 1 year after treatment initiation, the primary lesion was treated with a total of 72–78 Gy in 36–39 fractions of external irradiation as LRT. MDT was added with 15 fractions of total 37.5 Gy to this regimen whenever imaging studies showed lesions that may have been inadequately treated for PCa in patients with recurringly elevated PSA levels or pain caused by metastases. After LRT, ADT (alone) was continued in all patients. The patients were also evaluated for serum PSA and testosterone levels every 3 months, and imaging studies were performed every 6 months.

## 3 Endpoints and Statistical Analysis

The primary endpoint of this study was PFS. The secondary endpoints were OS, cancer‐specific survival, response rate, and adverse events. Progression was defined as PSA progression, the appearance of new lesions, or tumor regrowth on imaging studies (as assessed by the Response Evaluation Criteria in Solid Tumors) [11]. PSA progression was defined as an increase in PSA levels of ≥ 25% from the previous measurement and > 2.0 ng/mL from the nadir, with testosterone levels of < 50 ng/dL. Adverse events were evaluated according to the Common Terminology Criteria for Adverse Events, version 5.0. Data analysis was performed using JMP Pro 16 software (SAS Institute Inc., Cary, NC, USA). Continuous variables are expressed as median and interquartile range (IQR), while categorical variables are presented as frequency and percentage. Oncological outcomes were assessed from the date of pharmacological treatment initiation and analyzed using the Kaplan–Meier method. All *p*‐values were two‐tailed, and *p* < 0.05 was considered statistically significant.

## 4 Results

**FIGURE 1:**
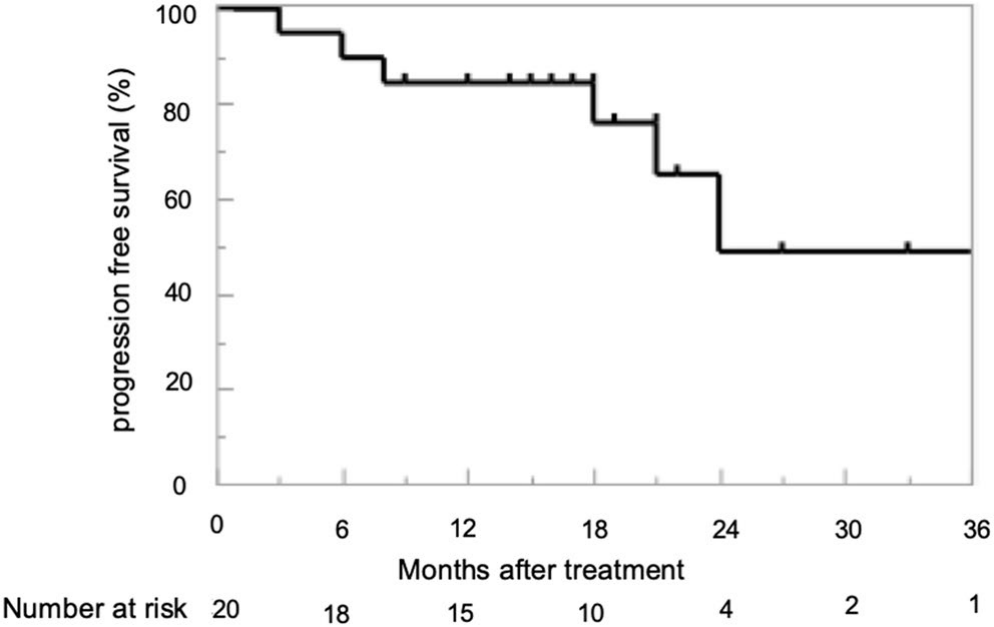
The progression‐free survival (PFS) of the enrolled patients was evaluated using Kaplan–Meier method. The one‐year PFS rate and median PFS were 84.4% and 18 months, respectively.

Enrollment in the study began in December 2021; 20 patients have been registered by May 2024, of whom 11 were followed up until May 2025 (Table 1). The median follow‐up period has been 18.0 months thus far (IQR, 14.0–25.8 months). At enrollment, the median patient age was 74 years (IQR, 72–79 years), PSA level was 273 ng/mL (94–663 ng/mL), and Bone Scan Index value was 2.29 (1.11–6.19). The median PSA levels were 0.58 ng/mL (0.20–12.0 ng/mL) at 3 months after treatment initiation, and 0.17 ng/mL (0.04–1.22 ng/mL) at 12 months. At 3 months after initiation, 17 of the enrolled patients (85.0%) had PSA reductions of ≥ 90% versus enrollment. Over the follow‐up period, PSA levels were maintained at < 0.2 ng/mL in 10 patients (50.0%). Seven patients (35.0%) were diagnosed with PSA progression, although no progression was observed on imaging. To date, none of the patients has died of PCa. The 1‐year PFS rate is 84.4% thus far, and the rPFS rate was 100% (median PFS, 18.0 months; Figure 1). Only three of the patients (15%) received MDT for cancer pain. The medication‐related adverse events reported thus far include grade 2 fatigue in three patients and grade 2 hepatic disorder, rash, and drug‐induced osteonecrosis of the jaw in one patient. No LRT‐related complications have been observed. The enzalutamide dose was reduced to 120 mg in three patients (15.0%) because of their older age, poor performance status in two patients, and a drug‐induced rash in one patient.

**TABLE 1:** Patient characteristics and early oncological outcomes.

No	Age	iPSA (ng/mL)	cT	N	M	GG	M‐site	BSI	PSA/BSI	Hot spot	LT (Gy)	MDT	3Mo PSA	12Mo PSA	BCR	Time to BCR (Mo)	Follow (Mo)
1	63	463	4	1	1b	4	B/Ly	1.61	288	20	72	−	0.38	0.38	+	21	43
2	80	5044	4	0	1b	3	B	11.6	435	122	66	−	18.1	1.0	+	24	42
3	66	577	3b	0	1b	5	B	9.64	59.9	95	−	−	12.4	70.3	+	6	18
4	79	54	4	1	1b	4	B	2.52	21.4	10	78	−	0.73	0.06	−		22
5	82	110	4	1	1b	5	B/Ly	6.54	16.8	99	−	−	29.5		+	3	5
6	77	296	3b	0	1c	5	B/Lu	0.76	389	4	−	−	0.17	0.01	−		16
7	74	897	4	1	1b	5	B	0.9	997	9	66	+	0.17	0.17	+	37	41
8	74	81	3a	1	1b	5	B	1.41	57.4	17	72	−	0.01	0.01	−		133
9	87	670	3b	1	1b	4	B/Ly	3.67	183	24	−	−	68.1	5.52	+	8	15
10	75	166	3a	0	1b	5	B	2.05	81.0	17	72	−	0.11	0.03	−		27
11	77	641	4	0	1b	4	B	3.77	170	11			14.3	0.90	−		12
12	73	282	3a	0	1c	4	B/Lu	1.01	279	4			0.27		−		9
13	73	55	3b	0	1b	5	B	5.93	9.27	84	72		10.9	1.44	+	18	18
14	72	3331	4	1	1b	5	B	7.97	418	89	30	+	0.77	0.05	−		21
15	71	243	4	1	1b	5	B	1.48	164	14	74	−	10.5	1.95	−		18
16	73	3.64	3b	1	1b	5	B/Ly	1.45	2.51	12	−	+	0.06	0.51	−		19
17	77	264	4	1	1b	5	B	5.81	45.4	43	−	−	0.58	0.02	−		15
18	61	88	4	1	1c	4	B/Lu	0.92	95.7	7			0.35	0.1	−		14
19	88	127	4	1	1b	5	B/Ly	0.111	1144	4	−	−	0.52	0.12	−		17
20	73	712	3b	0	1c	4	B/Lu	6.27	114	44			0.58		−		3

Abbreviations: 12Mo PSA, PSA at 12 months after neoadjuvant hormonal therapy; 3Mo PSA, PSA at 3 months after neoadjuvant hormonal therapy; B, bone; BCR, biochemical recurrence; BSI, bone scan index; GG, grade group; LT, local radiotherapy for primary tumor; Lu, lung; Ly, lymph node; MDT, metastasis‐directed therapy; Mo, months; M‐site, metastatic site; PSA, prostate specific antigen; PSA, prostate specific antigen.

## 5 Discussion

To date, no prospective studies have reported the combination of ARSI, LRT, denosumab, and MDT for poly‐PCa. Therefore, the presentation of the interim results of this single‐arm prospective study is considered meaningful for establishing treatment strategies for poly‐PCa. ARSI has been reported to enhance the therapeutic effect of LRT due to its ability to cause DNA damage, as compared to conventional ADT [12]. Therefore, LRT performed on the primary tumor after ARSI administration may be a more beneficial treatment for patients with poly‐PCa. We are currently investigating a cohort of such cases; therefore, the efficacy of this combination therapy for improving oncological outcomes remains to be determined. In the ARCHES trial, which included 38.3% patients with low‐volume PCa, the 1‐year PFS rate for ADT and ARSI was ~92%, with an rPFS rate of ~85% [13]. A similar observation was reported in the ENZAMET trial, which reported both 1‐year PFS and rPFS rates to be ~88% and ~90%, respectively, in a cohort of patients among whom 47% exhibited low‐volume PCa [1]. In the PEACE‐1 trial, which evaluated a triplet therapy comprising ADT, ARSI, and LRT, the 1‐year rPFS rate was ~88% in a patient cohort that included 43.2% with low‐volume disease [14]. A study of real‐world data on patients who were treated with both ADT and ARSI, among whom 31.1% had low‐volume PCa, reported a 1‐year PFS rate of ~85% [15]. Although direct comparisons with our protocol are challenging, these findings suggest that comparable outcomes may be achievable even in patients with poly‐metastatic PCa. Although all our patients achieved reductions in their PSA levels during our study thus far, the number of patients who experienced PSA progression has been low. In contrast, patients whose PSA levels have not decreased sufficiently with this treatment and who have experienced increases during the early period are expected to have early rPFS or progression to CRPC. We will continue to closely monitor these patients. The prognostic significance of PSA dynamics in mCSPC treated with ARSI remains to be elucidated. However, a minimum PSA value of < 0.2 ng/mL has been reported to represent a potential surrogate predictor of OS [16].

The present study is subject to several potential limitations. Regarding MDT, the number of patients who met the eligibility criteria for MDT in this study was limited, potentially causing an underestimation of its effectiveness. Moreover, given that this study uses a single‐arm design, it is not possible to compare the treatment outcomes of patients with PCa who received the current standard therapy with those who received our treatment regimen. Therefore, our study does not provide conclusive evidence regarding the efficacy of the treatment method used. Notably, this ongoing study is prospectively investigating a multimodal treatment strategy for poly‐PCa, including LRT. As a relatively large number of patients have already achieved sufficient reductions in PSA levels and there have been no cases of radiological progression, this treatment strategy is expected to improve the oncological outcomes of patients with poly‐PCa. Additionally, we are confident that reporting the interim results of this trial will contribute to the treatment of poly‐PCa. In the future, the number of registered cases will be increased, and the observation period will be extended until December 2027, with no additional extensions, and provide more comprehensive reports on this promising new treatment approach.

## Author Contributions

**Makoto Kawase:** conceptualization (equal), data curation (equal), formal analysis (equal), investigation (equal), methodology (equal), resources (equal), writing – original draft (equal). **Kota Kawase:** data curation (equal), resources (equal). **Yuki Tobisawa:** data curation (equal), investigation (equal), resources (equal). **Koji Iinuma:** data curation (equal), resources (equal). **Keita Nakane:** data curation (equal), resources (equal). **Takuya Koie:** conceptualization (equal), methodology (equal), project administration (equal), writing – review and editing (equal).

## Ethics Statement

Approval of the research protocol by an Institutional Review Board of Gifu University: 2021‐B041.

## Consent

This study obtained consent for all enrolled patients. The details of this study can be found at UMIN000054497.

## Conflicts of Interest

The authors declare no conflicts of interest.

## Data Availability

Data and materials are provided in this paper.
